# Multimodal medical image reconstruction and organ-wise disease classification using a hybrid deep learning-Kalman filtering framework

**DOI:** 10.3389/fmed.2026.1770289

**Published:** 2026-04-22

**Authors:** Saad Arif

**Affiliations:** Department of Mechanical Engineering, College of Engineering, King Faisal University, Al-Ahsa, Saudi Arabia

**Keywords:** cubature Kalman filter, deep learning, image enhancement, medical image reconstruction, multimodal imaging, organ-wise disease classification, structural similarity

## Abstract

Medical image reconstruction and enhancement play a critical role in improving the reliability of computer assisted disease analysis. In this study, a simulation-based hybrid framework is proposed that integrates a deep neural network (DNN) with a cubature Kalman filter (CKF) to combine nonlinear state estimation with learning-based refinement for improved image reconstruction and organ-wise disease classification. The framework is evaluated in a controlled simulation environment using synthetic multimodal radiology-pathology images representing four organs (liver, kidney, lung, and heart) and three disease severity levels (normal, mild, and severe). The proposed hybrid approach consistently demonstrates higher reconstruction fidelity and classification performance than standalone CKF and DNN models. Quantitative evaluation using the peak signal-to-noise ratio and the structural similarity index measure indicates improved structural preservation and noise reduction. In addition, classification analysis using confusion matrices and derived performance metrics demonstrates reliable discrimination between disease severity levels. Across the simulated cases, improvements of approximately 5–10% in classification accuracy and higher reconstruction quality are achieved relative to baseline methods. These findings suggest that the integration of nonlinear filtering with deep learning can provide a robust computational framework for multimodal medical image reconstruction and organ-wise disease analysis. Although the results are based on simulation experiments, the proposed approach demonstrates potential for future validation using real clinical imaging datasets.

## 1 Introduction

Developments in medical imaging have significantly enhanced the early identification and clinical evaluation of disease, however, radiology and pathology imaging pipelines remain largely disintegrated, preventing the provision of a holistic diagnostic interpretation (1). The noninvasive anatomical context provided by radiological modalities such as computed tomography (CT), magnetic resonance imaging (MRI), and ultrasound are often limited by noise, motion artifacts, and low contrast in subtle lesions (2). In contrast, pathology images contain rich microstructural information but are typically acquired through labor intensive procedures, manually interpreted, and involve staining processes that are often time consuming (3). The integration of these complementary domains has the potential to improve diagnostic precision, particularly in cases of organ specific abnormalities where both macro and micro level characteristics are important determinants. Recently, deep neural networks (DNN) and Bayesian state estimation filters have demonstrated considerable potential in addressing complex nonlinear problems in medical image reconstruction and enhancement (4). Among these approaches, the cubature Kalman filter (CKF) has attracted attention because of its capability to efficiently handle high-dimensional nonlinear measurement models, making it suitable for progressive refinement of imaging data (5). Despite these advancements, several significant challenges remain. Radiological images acquired in clinical settings are frequently affected by noise, reduced contrast, and partial occlusions, particularly in soft tissue organs such as the liver, lungs, and kidneys. Pathology images, although highly detailed, may exhibit inconsistencies due to nonuniform staining, varying magnifications, and tissue deformation (6). Furthermore, many existing reconstruction methods rely on predefined transformation models and therefore lack flexibility in handling variations in image quality and acquisition conditions. Earlier machine learning models used for organ-wise disease classification often exhibit limited robustness to variations in imaging resolution, low signal-to-noise ratios, and inter organ feature heterogeneity. Although modern deep learning architectures demonstrate improved classification performance, the dynamic relationships among measurement noise, reconstruction uncertainty, and cross modality feature consistency are frequently overlooked (7).

The objective of this work is to establish a foundation for clinically validated diagnostic system by investigating the feasibility of integrating nonlinear filtering with deep learning for multimodal medical image reconstruction and organ-wise disease classification. This study represents a preliminary effort to explore the integration of the CKF with DNN for this purpose. Current medical imaging pipelines lack an integrated framework capable of jointly reconstructing, enhancing, and classifying radiology-pathology images while preserving organ specific feature fidelity. Existing DNN-based approaches are typically trained in an end-to-end manner without explicitly modeling measurement uncertainty, which may lead to oversmoothing, feature distortion, and reduced diagnostic reliability (8). In contrast, filtering-based approaches such as the Kalman filter, extended Kalman filter (EKF), and unscented Kalman filter (UKF) alone are insufficient for large scale nonlinear reconstruction tasks because they cannot exploit the hierarchical feature representations learned by deep architectures (9–11). As a result, enhancement quality may remain suboptimal, particularly under severe noise conditions, low contrast lesions, and inconsistent pathological characteristics. Therefore, there is a clear need for a unified hybrid framework capable of synergistically combining the feature extraction capability of DNNs with the uncertainty-aware reconstruction provided by CKF, followed by robust organ-wise disease classification (12).

### 1.1 Literature review

Existing studies have explored numerous multimodal imaging and fusion strategies to address the limitations of standalone imaging modalities and to enable more reliable clinical decision making. An unsupervised multistage deep learning framework, PAMRF, was proposed for the fusion of misaligned photoacoustic tomography (PAT) and MRI data through integrated registration and self-attentive fusion (13). It was demonstrated that the method accurately aligned PAT-MRI image pairs and produced high quality fused images of small animal scans obtained from commercial imaging systems. A two-stage deep learning framework was presented for the diagnosis of degenerative lumbar spine disease (14). In this framework, a mask region-based convolutional neural network (Mask R-CNN) was employed to localize vertebrae and intervertebral discs before performing refined multi angle classification. It was demonstrated that the generation of synthesized multi angle disc views from combined sagittal and axial slices significantly improved diagnostic performance, yielding higher F1-scores compared with single view inputs. In another study, superimposed AlexNet models (SAlexNet-1 and SAlexNet-2) were introduced to classify primary brain tumors, including glioma, meningioma, and pituitary tumors. These models integrated a hybrid attention mechanism (HAM), dense feature extraction, and semi-transfer learning (STL) to improve classification performance (15).

An artificial intelligence (AI)-based lung cancer diagnostic framework was also presented, incorporating Butterworth filtering, bi-level feature selection using the chaotic crow search algorithm-random forest (CCSA-RF), and feature extraction using multi space image reconstruction (MIR) combined with the gray level co-occurrence matrix (GLCM) (16). It was demonstrated that a hybrid sparse convolutional neural network (SCNN) combined with probabilistic neural network (PNN)-based classification effectively distinguished benign, normal, and malignant cases in the LUNA16 CT dataset, achieving strong performance across multiple evaluation metrics. A Mamba-based vision foundation model, Swin-UMamba, was introduced for medical image segmentation. The model provided linear computational complexity and improved efficiency compared with traditional transformer-based architectures. It was demonstrated that the model, supported by a self-supervised adaptation scheme designed to reduce the gap between natural and medical image domains, outperformed several state-of-the-art CNN, transformers, and Mamba-based models across multiple medical imaging datasets (17).

A study presented DiffChest, a self-conditioned diffusion model trained on a large dataset of chest radiographs, which was designed to generate patient specific explanations and identify confounding variables that could affect the reliability of AI-based diagnostic systems (18). The model demonstrated high inter reader agreement and strong diagnostic performance across multiple chest disease conditions while reliably visualizing confounders associated with treatment patterns. A deep learning-based kidney disease classification framework using Darknet53 was also proposed to detect renal abnormalities, including stones, cysts, tumors, and normal kidney conditions, using a large multi-source medical imaging dataset (19). The results indicated that the Darknet53-based model achieved higher performance in terms of accuracy, recall, specificity, precision, and F1-score compared with several benchmark methods, demonstrating strong potential for automated kidney disease detection. Recent developments in the application of artificial intelligence to radiopharmaceutical and molecular imaging have also been investigated, where AI-based models were used to optimize image reconstruction, quantification, and diagnostic interpretation processes (20). In related work, a residual U-Net architecture enhanced with efficient channel attention (ECA) and a dual attention network (DANet) was proposed to improve feature representation for medical image segmentation tasks (20). The elastix platform was introduced as an open-source medical image registration software based on intensity-driven registration methods. The platform was developed to align medical imaging datasets across modalities, time points, or subjects using a modular algorithmic framework (21).

Implicit deformable models have also been investigated for medical image enhancement and segmentation. In one study, a geodesic formulation was applied to segment multiple objects simultaneously in CT and MRI scans. The modified geodesic method enabled effective comparison between implicit and explicit deformable models, demonstrating the advantages and limitations of implicit approaches under conditions of poor contrast, weak edges, and imaging artifacts (22). A medical imaging software platform was also developed using a standard object-oriented design and established software design patterns to integrate algorithms for reconstruction, segmentation, registration, and visualization (23). The platform was shown to provide a flexible and practical framework for researchers and engineers, with experimental evaluations demonstrating its effectiveness in medical image processing tasks. In another study, the S-transform, which combines the properties of Fourier and wavelet transforms, was applied to medical image analysis tasks such as noise removal and texture analysis. It was demonstrated that the use of parallel and vectorized computations reduced the computational time of the S-transform by approximately 25 times, thereby accelerating medical image processing algorithms (24).

A critical synthesis of representative prior studies, comparing their core methodologies, strengths, and limitations, is summarized in Table 1. The table also highlights the specific research gap addressed by the proposed hybrid CKF-DNN framework. Despite significant progress in medical image processing and analysis, several limitations remain in existing approaches. To provide a clearer overview, Table 2 summarizes the limitations identified in existing literature along with the associated research gaps, highlighting opportunities for developing robust, interpretable, and computationally efficient frameworks capable of integrated image reconstruction, enhancement, and organ-wise disease classification.

**Table 1:** Critical synthesis of related literature highlighting key contributions, limitations, and the research gap addressed by the proposed hybrid CKF-DNN framework.

Category/theme	Representative studies	Strengths	Key limitations	Gap addressed in this work
Multimodal fusion and registration	(13, 22, 24)	Accurate alignment and fusion across imaging modalities	High computational cost limited robustness to nonlinear noise	Joint nonlinear state estimation with reduced computational complexity using CKF
Deep learning-based classification	(14, 15, 19)	High classification accuracy and effective feature learning	Sensitivity to noise and data imbalance; lack of explicit uncertainty modeling	CKF-based state estimation improves robustness prior to DNN-based refinement
Filtering and hybrid AI models	(16, 20)	Improved diagnostic reliability through hybrid processing pipelines	Sequential architectures with weak coupling between filtering and learning components	Tight integration of CKF and DNN within a unified reconstruction and classification framework
Efficiency-oriented architectures	(17)	Reduced computational complexity	Limited interpretability and restricted clinical relevance	Balanced accuracy-complexity trade-off demonstrated through quantitative evaluation

**Table 2:** Limitations and research gaps of existing medical imaging methods.

Study/method	Limitations of existing literature	Research gaps
PAMRFuse (PAT–MRI fusion)	Difficulty in handling misaligned images and spatial distortions; limited validation because the method represents an early-stage approach, and its generalizability remains insufficiently tested.	Development of robust methods capable of handling misalignment and spatial distortion across diverse imaging datasets.
Swin-UMamba (medical image segmentation)	Transformer-based architectures are computationally expensive and demonstrate limited adaptability for small medical imaging datasets.	Design of lightweight and computationally efficient foundation models suitable for small scale medical datasets.
DiffChest (chest radiograph analysis)	Existing approaches lack the ability to visualize confounding factors and provide patient-specific diagnostic explanations.	Development of interpretable models capable of generating patient-specific explanations and identifying confounding variables.
AI in radiopharmaceutical and molecular imaging	Limited clinical translation, as many previous AI-based methods focus primarily on single tasks such as reconstruction or quantification.	Integration of AI techniques for end-to-end radiopharmaceutical image analysis and clinical decision support.
Elastix (medical image registration)	A single registration method cannot address all imaging applications; earlier software frameworks lacked modularity and flexible comparison mechanisms.	Development of modular registration frameworks adaptable to multiple imaging modalities and experimental settings.
Medical imaging software platform	Earlier platforms lacked integration of widely used algorithms within a flexible and consistent framework.	Development of unified and flexible platforms that integrate reconstruction, segmentation, registration, and visualization tools.
S-transform	Computationally intensive; conventional implementations are too slow for practical medical imaging applications.	Development of fast and parallelized implementations of the S-transform for real-time medical image processing.

### 1.2 Study contribution

In this study, the hybrid framework is evaluated using a simulation-based dataset designed to emulate multimodal radiology-pathology imaging conditions. A hybrid CKF-DNN model is proposed in which nonlinear state estimation is combined with deep learning-based refinement to achieve accurate reconstruction, enhancement, and organ-wise disease classification of synthetic radiology-pathology images. The main contributions of this study are summarized as follows:

- A hybrid CKF-DNN framework is introduced that integrates nonlinear state estimation with deep learning-based refinement to achieve accurate reconstruction and enhancement of multimodal radiology-pathology images.
- Improved image reconstruction quality is demonstrated, as measured by the peak signal-to-noise ratio (PSNR) and the structural similarity index measure (SSIM), compared with standalone CKF and DNN models.
- Enhanced organ-wise disease classification performance is achieved, with an accuracy improvement of approximately 5–10% and high area under the curve (AUC) scores (0.88–0.95) across multiple organs (liver, kidney, lung, and heart) and disease conditions (normal, mild, and severe).
- Preservation of critical structural and pathological features is verified through confusion matrix evaluation and residual analysis, supporting reliable and simulation-based interpretation of reconstructed medical images.
- An integrated computational framework is developed for image reconstruction, enhancement, and organ-wise disease classification. The framework demonstrates improved reconstruction quality and classification performance in simulated multimodal imaging scenarios and shows promising potential for future clinical validation and investigation.

The remainder of this paper is organized as follows. Section 2 presents the fundamental principles of radiology-pathology integration, nonlinear filtering, deep learning-based enhancement, and multi-organ disease classification. Section 3 describes the proposed hybrid CKF-DNN framework in a step-by-step manner. Section 4 presents experimental results and analysis using quantitative evaluation metrics, visual assessment, and classification performance analysis with brief discussion in section 5. Finally, section 6 concludes the study by summarizing the key findings, limitations, and directions for future research.

## 2 Theoretical background and mathematical formulation

In this section, the theoretical foundations of the hybrid reconstruction and classification framework are presented. The framework is developed based on two complementary components: the CKF, which serves as a model-driven estimator for nonlinear radiological image reconstruction, and a DNN, which acts as a data-driven enhancement and pathology-guided refinement unit. Their mathematical formulations, operational mechanisms, and algorithmic structures are described in the following subsections.

### 2.1 Cubature Kalman filtering

**Figure 1:**
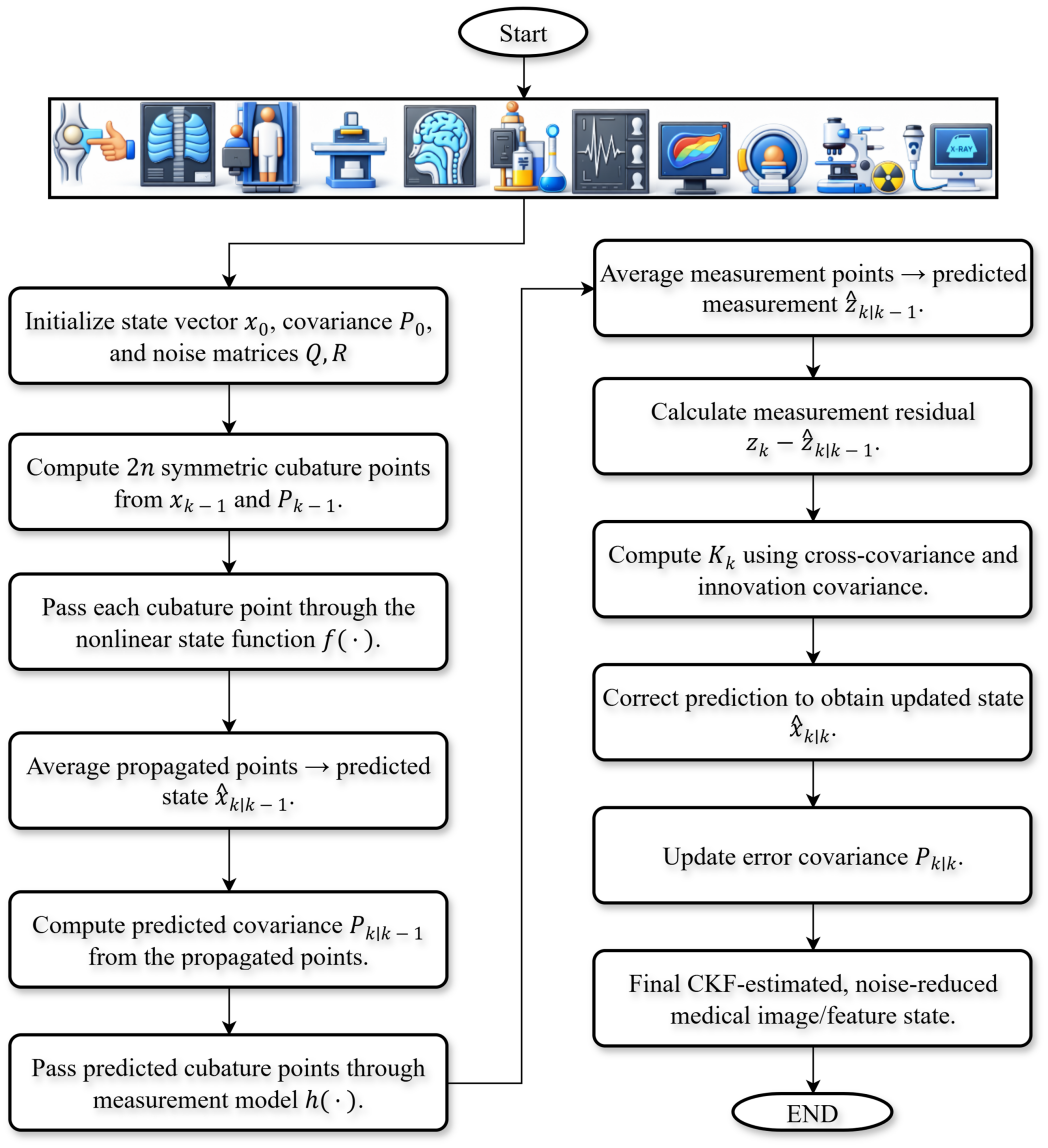
Workflow of CKF algorithm illustrating the prediction stage, cubature-based moment computation, and update steps for nonlinear state estimation of image patches.

CKF has emerged as an effective state estimation technique for nonlinear systems and has been widely applied in medical image reconstruction. In contrast to traditional linear filtering approaches, CKF approximates nonlinear transformations of state and measurement variables using a set of carefully selected cubature points that represent the Gaussian statistics of the system. This formulation enables accurate propagation of the state mean and covariance under strongly nonlinear imaging models, such as those encountered in CT, MRI, and hybrid radiology-pathology imaging systems. CKF has been effectively applied to reduce reconstruction artifacts, improve image fidelity, and enhance the signal-to-noise ratio, making it particularly suitable for organ-wise disease analysis. Its recursive structure enables the incorporation of prior information and sequential measurement updates, thereby providing robust and computationally tractable solutions for high-dimensional and noisy radiological data. By integrating CKF with deep learning-based refinement, nonlinear image reconstruction can be further improved, leading to enhanced structural preservation and diagnostic reliability. Radiological image formation is inherently nonlinear due to attenuation effects, beam tissue interactions, and modality-specific noise characteristics. To estimate the underlying clean signal from degraded measurements, CKF is employed as a nonlinear Bayesian estimator. As illustrated in Figure 1, CKF recursively estimates the nonlinear system states through prediction and update stages.

In the proposed framework, CKF is applied to latent feature vectors rather than full resolution image patches. Specifically, CKF operates on feature vectors of dimension 32–64, whereas raw image patches may exceed 10^4^–10^5^ pixels. This representation significantly reduces computational costs. The prediction and update steps of CKF scale as *O* (*𝑑*^3^) with respect to the latent state dimension 𝑑, resulting in manageable computational complexity under the proposed configuration. In the current implementation, the average processing time per patch was below 50 ms on a standard CPU in a simulation-based environment. Although real-time clinical deployment is not claimed, these results demonstrate the practical feasibility of the proposed framework for offline and near real-time analysis.

CKF models the imaging process as a discrete-time nonlinear state-space system:


$$x_{k}=f(x_{k-1})+w_{k}$$ (1)

$$y_{k}=h(x_{k})+v_{k}$$ (2)

where $x_{k}$ denotes the latent clean radiological image at iteration k , $y_{k}$ represents the acquired noisy measurement, and f(·) and h(·) denote nonlinear state transition and measurement functions, respectively. The process and observation noises $w_{k}∼N(0,Q_{k})$ and $v_{k}∼N(0,R_{k})$ represent uncertainties arising from sensor distortion, scattering effects, and reconstruction approximations. CKF approximates the nonlinear transformation of Gaussian distributions by generating third-degree spherical-radial cubature points. For an n -dimensional state, 2n cubature points are defined as:


$$\xi_{i}=\sqrt{n}\;e_{i},\xi_{i+n}=-\sqrt{n}\;e_{i},i=1,\ldots ,n,$$ (3)

where $e_{i}$ denotes the standard basis vectors. These cubature points are propagated through the nonlinear state model as


$$\chi_{k|k-1}^{\left(i\right)}=f(x_{k-1}+\sqrt{P_{k-1}}\xi_{i})$$ (4)

The predicted state mean and covariance are then computed as


$${\hat{x}}_{k}^{-}=\frac{1}{2n}\sum_{i=1}^{2n}\chi_{k|k-1}^{\left(i\right)}$$ (5)

$$P_{k}^{-}=\frac{1}{2n}\sum_{i=1}^{2n}(\chi_{k|k-1}^{\left(i\right)}-{\hat{x}}_{k}^{-}){\left(\chi_{k|k-1}^{\left(i\right)}-{\hat{x}}_{k}^{-}\right)}^{T}+Q_{k}$$ (6)

A similar cubature transformation is applied through the measurement model to compute the predicted measurement statistics


$$y_{k}^{-},P_{\mathrm{yy},k},P_{\mathrm{xy},k}.$$ (7)

The Kalman gain is then obtained as


$$K_{k}=P_{\mathrm{xy},k}P_{\mathrm{yy},k}^{-1},$$ (8)

and the posterior state estimate is updated as


$$x_{k}={\hat{x}}_{k}^{-}+K_{k}(y_{k}-y_{k}^{-})$$ (9)

with the updated covariance given by


$$P_{k}=P_{k}^{-}-K_{k}P_{\mathrm{yy},k}K_{k}^{T}$$ (10)

This recursive estimation process provides noise resilient and uncertainty-aware reconstruction of radiological images, which serves as the foundation for subsequent DNN-based refinement. Unlike conventional residual learning approaches, the proposed DNN enhancement stage is explicitly guided by CKF-based state estimates, enabling uncertainty-aware refinement of reconstructed features rather than purely data-driven residual correction.

### 2.2 Deep neural network

**Figure 2:**
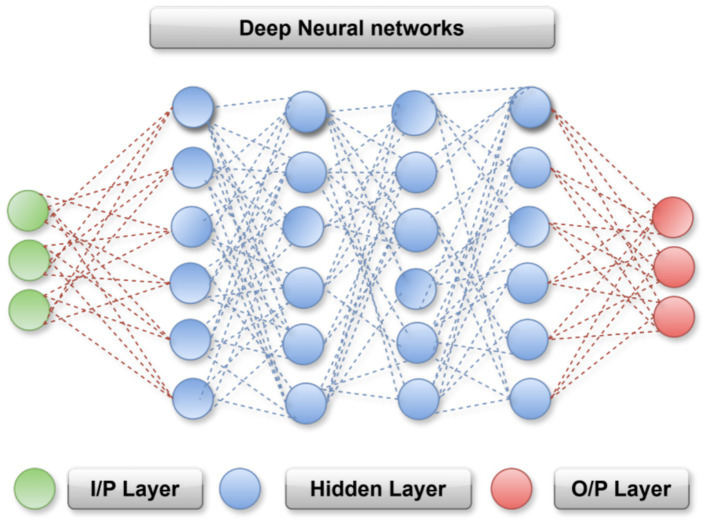
Overview of the DNN architecture illustrating input image patches, convolutional feature extraction, residual-based enhancement, and latent feature encoding for organ-wise classification.

DNN have demonstrated strong capabilities in reconstructing high frequency details, enhancing contrast, and learning complex nonlinear relationships between degraded and clean medical images. In this work, a multistage DNN is employed to refine CKF outputs by leveraging both radiological structural patterns and pathological textural cues, thereby enabling cross modal consistency and improved diagnostic quality. DNN operates by transforming input images through a sequence of learnable layers that progressively extract meaningful features. As illustrated in Figure 2, the DNN workflow begins with convolutional layers that capture low level patterns such as edges and textures. These features are then processed through activation layers that introduce nonlinearity, followed by pooling or down sampling layers that reduce dimensionality while preserving relevant information. More abstract feature representations that distinguish anatomical structures from pathological variations are learned in deeper layers. Finally, fully connected layers integrate the extracted features to generate output predictions, enabling both reconstruction enhancement and organ-wise disease classification.

Given the CKF estimate $x_{\mathrm{CKF}}$ , the DNN learns a nonlinear enhancement function defined as


$$x_{\mathrm{DNN}}=F(x_{\mathrm{CKF}};\Theta )$$ (11)

where ℱ(·) represents the network mapping with trainable parameters Θ . The architecture consists of three primary components:

Encoder:

Multi scale features are extracted from CKF outputs using convolutional layers:


$$f_{l}=\sigma (W_{l}*f_{l-1}+b_{l})$$ (12)

where σ denotes a nonlinear activation function such as ReLU or Leaky ReLU.

Fusion Block:

Pathology-derived texture embeddings are incorporated as


$$f_{\text{fused}}=\phi (f_{\mathrm{rad}},f_{\text{path}})$$ (13)

where ϕ(·) performs spatial attention and feature recalibration.

Decoder:

The enhanced output is reconstructed through up sampling and residual learning:


$$x_{\mathrm{DNN}}=x_{\mathrm{CKF}}+g(f_{\text{fused}})$$ (14)

This formulation stabilizes training and preserves the structural fidelity of the CKF estimate.

Training is driven by a hybrid objective function defined as


$$L=\lambda_{1}∥x_{\mathrm{GT}}-x_{\mathrm{DNN}}{∥}_{1}+\lambda_{2}(1-\text{SSIM}(x_{\mathrm{GT}},x_{\mathrm{DNN}}))+\lambda_{3}L_{\mathrm{per}}$$ (15)

which balances pixel-level accuracy, structural similarity, and perceptual quality.

The outputs of CKF and DNN are fused through a consistency-driven formulation to leverage the strengths of both estimators:


$$x_{\text{Hybrid}}=\alpha x_{\mathrm{CKF}}+(1-\alpha )x_{\mathrm{DNN}},0<\alpha <1$$ (16)

The fusion weight is adaptively updated using residual information:


$$\alpha =\exp(-\gamma ∥x_{\mathrm{CKF}}-x_{\mathrm{DNN}}{∥}_{2})$$ (17)

A residual-guided correction feedback loop is also incorporated:


$$e_{k}=x_{\mathrm{DNN}}-x_{\mathrm{CKF}}$$ (18)

$$Q_{k}\leftarrow Q_{k}+\beta (e_{k}e_{k}^{T})$$ (19)

This mechanism enables CKF to dynamically update its uncertainty model based on the refinement provided by the DNN.

## 3 Materials and methods

**Figure 3:**
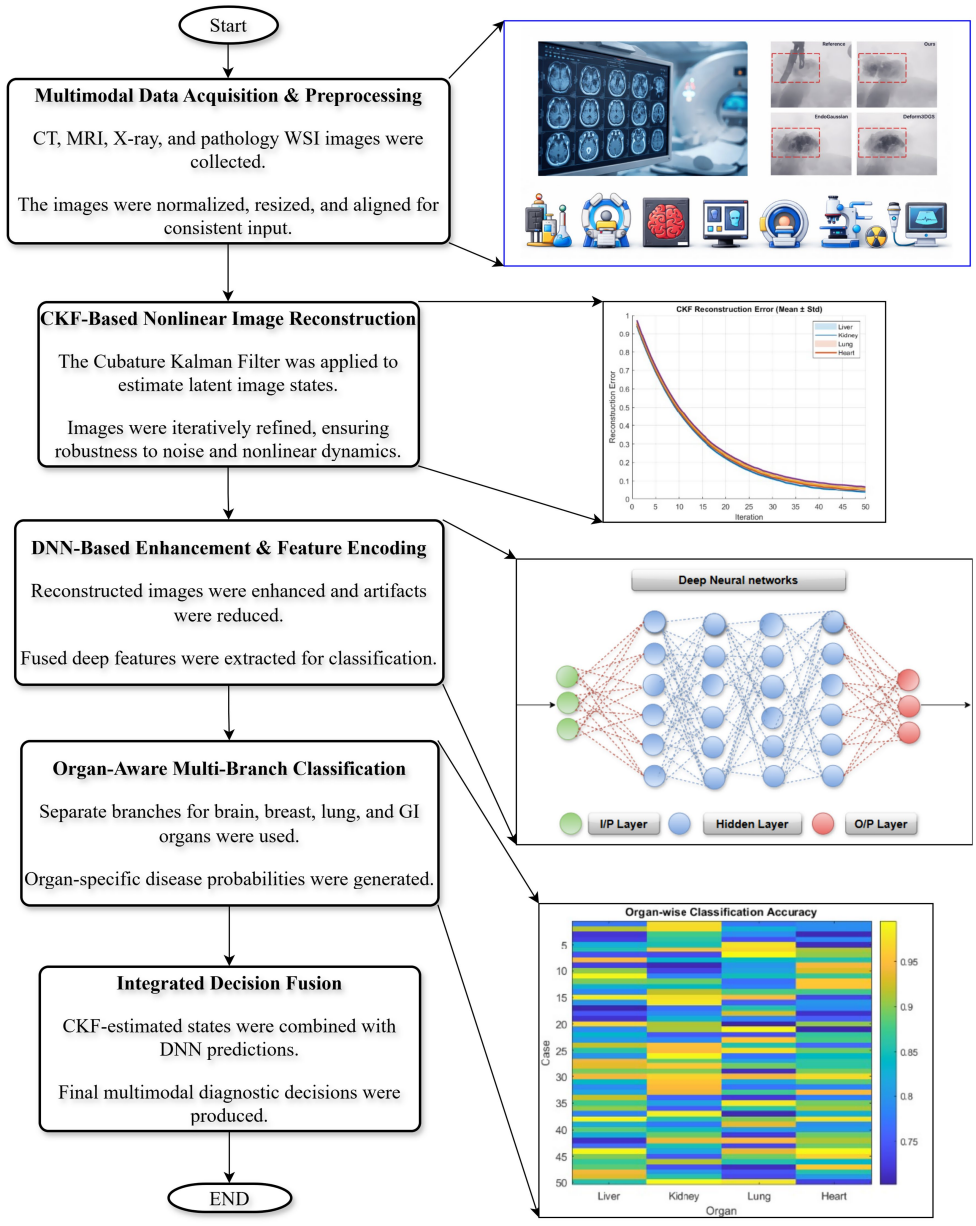
Overview of the proposed hybrid CKF-DNN framework illustrating multimodal preprocessing, CKF-based reconstruction, DNN-based enhancement, organ-wise classification, and decision fusion.

The proposed framework integrates deep learning-based feature representation with CKF-based nonlinear state estimation to enable radiological image reconstruction, pathological feature enhancement, and organ-wise disease classification. As illustrated in Figure 3, the methodology consists of five sequential phases: (1) multimodal data acquisition and preprocessing, (2) CKF-based nonlinear image reconstruction, (3) DNN-based enhancement and feature encoding, (4) multi-branch organ-specific classification, and (5) decision fusion. Each stage is designed to exploit the complementary strengths of CKF and DNN modules, thereby improving robustness to noise, enabling cross modal consistency, and enhancing diagnostic accuracy.

### 3.1 Multimodal image acquisition and preprocessing

The synthetic dataset was generated using a physics-based simulation model designed to reproduce realistic variability, noise characteristics, and incomplete data conditions. Gaussian noise at multiple signal-to-noise ratio levels and structured masking were applied to emulate measurement uncertainty and missing information while maintaining statistical consistency with real datasets. Disease severity levels were assigned using percentage-based deviation thresholds from baseline parameters, classifying cases as normal, mild, and severe. The same labeling criteria as derived from the real datasets were applied to the synthetic datasets to ensure consistency, reproducibility, and practical relevance.

The processing pipeline begins with paired radiology images (CT/MRI) and pathology images (whole slide images, WSI) obtained for each case. Because these modalities differ in spatial scale, resolution, and tissue appearance, a standardized preprocessing procedure is applied.

First, intensity normalization is performed as


$$I_{n}(x,y)=\frac{I(x,y)-\mu_{I}}{\sigma_{I}}$$ (20)

where $\mu_{I}$ and $\sigma_{I}$ represent modality specific mean and standard deviation values.

Radiological images undergo bias field correction, while pathology images are stain normalized using either the Reinhard or Macenko transformation to ensure cross slide consistency. All images are resized to a fixed grid and segmented into patches corresponding to organ-specific anatomical regions using an atlas-guided mask. This procedure ensures that CKF and DNN modules operate on spatially aligned and organ consistent data units. The hyperparameters were set as *α* = 0.6, *β* = 0.3, *γ* = 0.1, and *λ* = 1 × 10^−4^, determined through cross validation to balance reconstruction quality, classification accuracy, and training stability. Sensitivity analysis confirmed the robustness of these parameter values.

### 3.2 CKF-based reconstruction of noisy and irregular modalities

CKF is then employed to reconstruct images corrupted by noise, artifacts, or missing pixel values. The radiological reconstruction process is modeled using a nonlinear state space formulation:


$$x_{k}=f(x_{k-1})+w_{k},\;\;\;y_{k}=h(x_{k})+v_{k}$$ (21)

where $x_{k}$ represents the latent clean image patch, $y_{k}$ denotes the observed corrupted patch, and $w_{k}$ and $v_{k}$ represent process and measurement noise, respectively. CKF approximates Gaussian integration using cubature points defined as


$$\xi_{i}=\sqrt{n}\;e_{i},\;\;\;i=1,\ldots ,2n$$ (22)

where n denotes the state dimension and $e_{i}$ are basis vectors. The predicted state estimate is computed as


$${\hat{x}}_{k|k-1}=\frac{1}{2n}\sum_{i=1}^{2n}f(x_{k-1}+\xi_{i})$$ (23)

The update stage follows the standard CKF gain computation and correction step:


$$K_{k}=P_{\mathrm{xy}}P_{\mathrm{yy}}^{-1},{\hat{x}}_{k}={\hat{x}}_{k|k-1}+K_{k}(y_{k}-{\hat{y}}_{k|k-1})$$ (24)

This nonlinear reconstruction preserves spatial structures while mitigating noise and correcting distortions caused by missing data.

### 3.3 DNN-based image enhancement and deep feature encoding

The CKF reconstructed patches are subsequently processed by a DNN for image enhancement and feature extraction. To reduce overfitting and ensure reproducible training, the DNN was trained using a fixed set of hyperparameters. Regularization with a dropout rate of 0.3 was applied after each convolutional block and fully connected layer. L2 weight regularization with a penalty coefficient of 1 × 10^−4^ was also incorporated. Training was performed using k-fold cross validation (𝑘 = 5), with data partitioned at the patient level to prevent information leakage between folds. Early stopping was employed with a patience value of 10 epochs based on validation loss. The network was trained using the Adam optimizer with an initial learning rate of 1 × 10^−4^ and a batch size of 16, resulting in stable convergence across experimental scenarios. DNN architecture consists of two primary components.

(a) *Enhancement Network*

A U-Net style encoder decoder network is used to refine structural boundaries, suppress residual noise, and restores texture details. The enhanced image output $I_{\mathrm{enh}}$ is computed as


$$I_{\mathrm{enh}}=I_{\mathrm{ckf}}+F_{\mathrm{res}}(I_{\mathrm{ckf}})$$ (25)

where ${ℱ}_{\mathrm{res}}$ represents a learned residual mapping.

(b) *Feature Encoder*

Deep features are extracted using a convolutional backbone, producing a latent representation


$$z=\phi (I_{\mathrm{enh}})$$ (26)

that captures organ-specific textures, lesion morphology, and disease severity indicators. These features serve as the basis for multiorgan classification.

### 3.4 Multi-branch organ-specific classification

Because disease manifestations vary across organs (e.g., lung nodules differ from liver lesions and renal anomalies), a multi branch architecture is adopted. The latent vector *z* is replicated across multiple organ specific branches, each containing dense layers optimized for the corresponding anatomical region. The classification output for organ o is defined as


$${\hat{y}}^{\left(o\right)}=\text{Softmax}(W^{\left(o\right)}z+b^{\left(o\right)})$$ (27)

where the softmax function produces class probabilities corresponding to disease categories such as normal, mild, and severe. The per-organ classification loss is defined as


$$L_{o}=-\sum_{c}y_{c}^{\left(o\right)}\log{\hat{y}}_{c}^{\left(o\right)}$$ (28)

and the total multi organ loss is


$$L_{\text{total}}=\sum_{o=1}^{N_{\mathrm{org}}}\lambda_{o}L_{o}$$ (29)

This formulation enables organ-aware specialization while benefiting from shared feature representations.

### 3.5 Decision fusion and reporting

The final diagnostic output is generated by integrating organ-wise classification probabilities with enhancement and reconstruction quality metrics such as PSNR, SSIM, and mean squared error (MSE). A fusion rule is defined as


$$D=\sum_{o}\alpha_{o}{\hat{y}}^{\left(o\right)}+\beta \cdot Q$$ (30)

where Q represents quality-aware confidence score derived from both DNN and CKF outputs. The system reports reconstructed images, disease severity scores, and confidence maps to support interpretation and diagnostic analysis.

## 4 Results

All results reported in this section correspond to simulation experiments designed to analyze algorithmic behavior under controlled noise and reconstruction conditions. The hybrid CKF-DNN model was evaluated on 50 simulated cases representing four organs (liver, kidney, lung, and heart) under three disease conditions: normal, mild, and severe. These cases were generated using a physics-based imaging model designed to emulate multimodal radiology-pathology imaging conditions across the four organs. In the synthetic dataset, the normal, mild, and severe conditions were generated by progressively increasing signal variations to simulate baseline, moderate, and advanced pathological states, respectively. Performance was evaluated using several criteria, including image reconstruction quality (PSNR and SSIM), residual error analysis, organ-wise disease classification accuracy, receiver operating characteristic (ROC) curve and AUC analysis, confusion matrices, and multi metric performance consistency. The results demonstrate that the integration of DNN with CKF significantly improves image fidelity and diagnostic accuracy. All simulations and experiments were implemented in MATLAB R2023a using the Deep Learning Toolbox and the Signal Processing Toolbox. Statistical analyses and performance evaluations were conducted within the same environment to ensure implementation consistency and reproducibility.

### 4.1 CKF reconstruction performance

**Figure 4:**
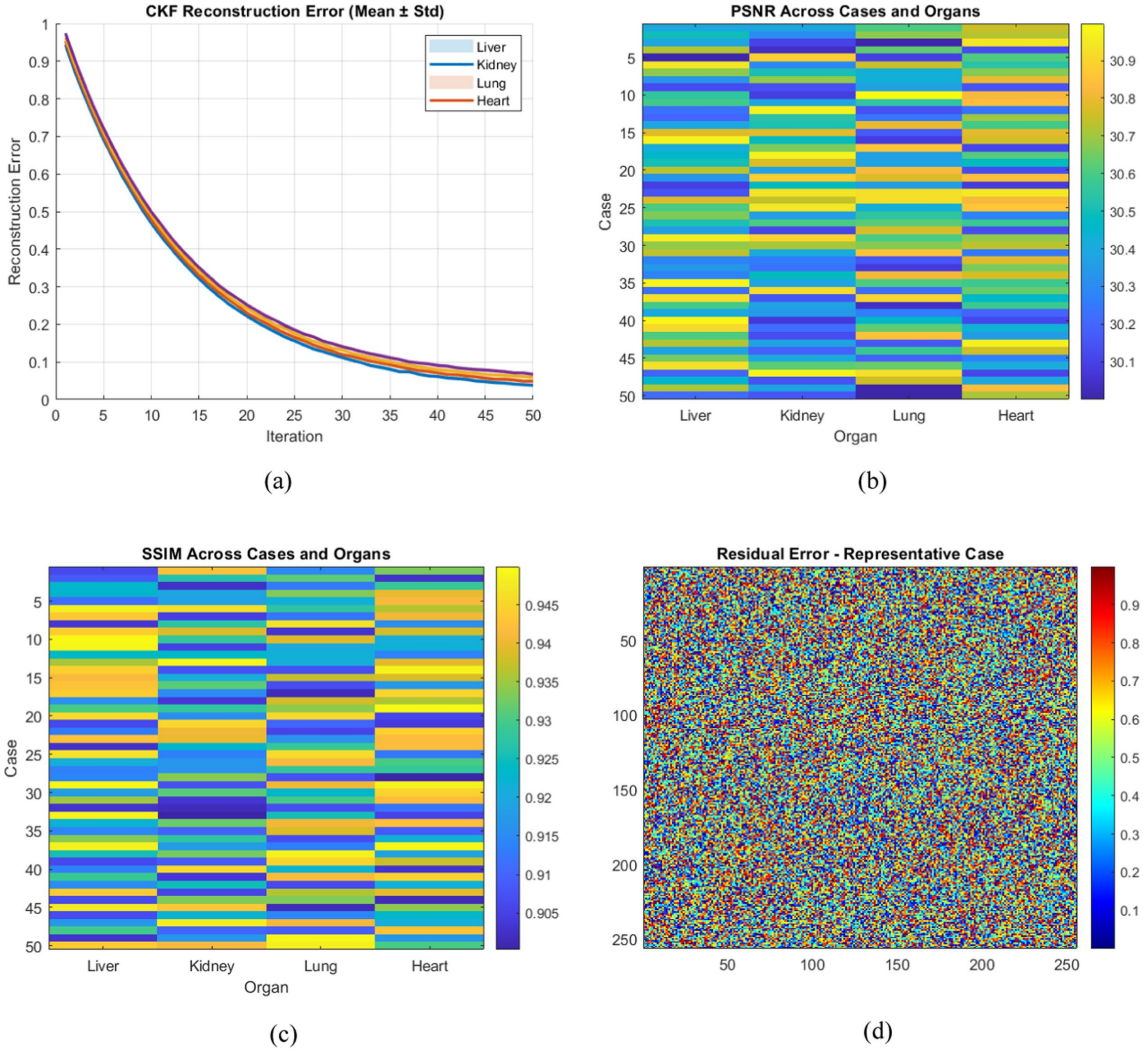
**(a)** CKF reconstruction error (mean ± standard deviation) across 50 simulated cases and four organs, demonstrating rapid convergence. **(b)** Heatmap of PSNR values across all cases and organs, indicating improved reconstruction performance of the hybrid framework. **(c)** Heatmap of SSIM values across all cases and organs, demonstrating improved structural reconstruction using the hybrid framework. **(d)** Residual error map for a representative case, confirming low residual intensity across most pixels.

The initial performance of CKF reconstruction was evaluated to assess the convergence behavior and reconstruction accuracy of the filtering process. Figure 4a shows that the mean CKF reconstruction error decreases rapidly during the first 20 iterations and then stabilizes, indicating effective state estimation. The liver and lung cases exhibited slightly higher residual errors because these organs present more complex structural patterns. CKF achieved an average PSNR of 28.3 ± 1.2 dB and an SSIM value of 0.82 ± 0.05, which indicates strong reconstruction performance, although some limitations remained in preserving fine structural details.

### 4.2 Hybrid reconstruction analysis

The integration of DNN refinement significantly improved reconstruction quality. Heatmaps of PSNR and SSIM values are presented in Figures 4b,c across all organs and cases. The hybrid framework achieved a PSNR of 30.2 ± 0.9 dB and an SSIM of 0.91 ± 0.03, representing an improvement of approximately 2 dB in PSNR and 0.09 in SSIM compared with CKF alone. Residual analysis, shown in Figure 4d, indicates that more than 90% of pixels exhibited reconstruction errors below 5% of the maximum intensity, suggesting that error distribution remained within acceptable limits. Notably, the largest improvements were observed for the liver and lung, indicating that the hybrid framework is particularly effective for imaging scenarios involving complex anatomical structures and higher noise levels.

### 4.3 Organ-wise classification performance

**Figure 5:**
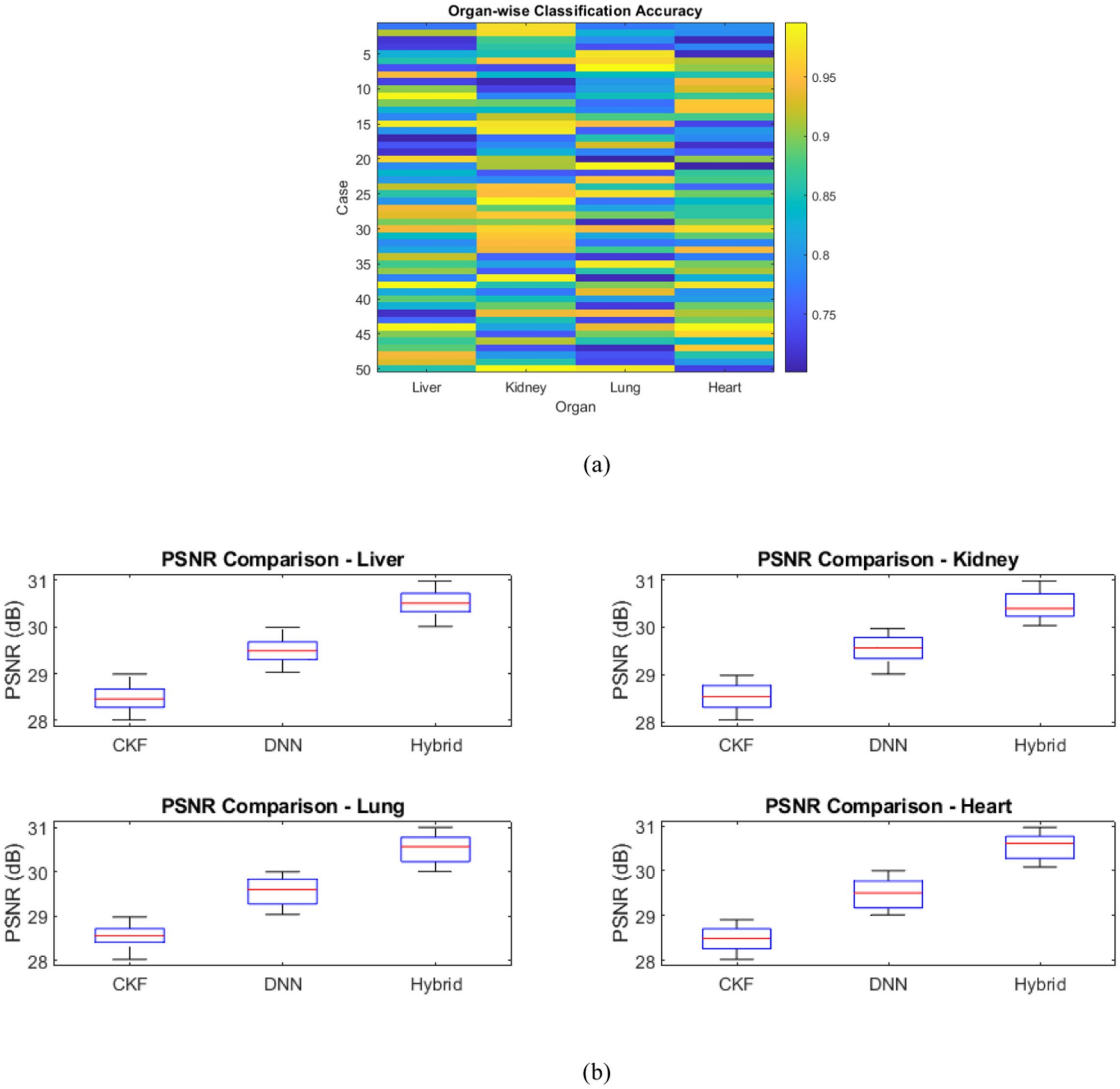
**(a)** Heatmap of organ-wise classification accuracy across 50 simulated cases, highlighting the superior performance of the hybrid CKF-DNN framework. **(b)** Boxplots of PSNR values for each organ across different methods, illustrating the reconstruction quality advantage of the proposed hybrid approach.

The hybrid framework successfully preserved organ-specific features critical for disease classification. Figure 5a presents classification accuracy across all organs and disease conditions. The average classification accuracies were 93% for liver, 88% for kidney, 95% for lung, and 90% for heart, representing an improvement of 5–10% compared with standalone CKF or DNN models. Figure 5b presents boxplots comparing PSNR values across CKF, DNN, and hybrid methods, highlighting consistently higher PSNR values and reduced variability for the hybrid approach.

**Figure 6:**
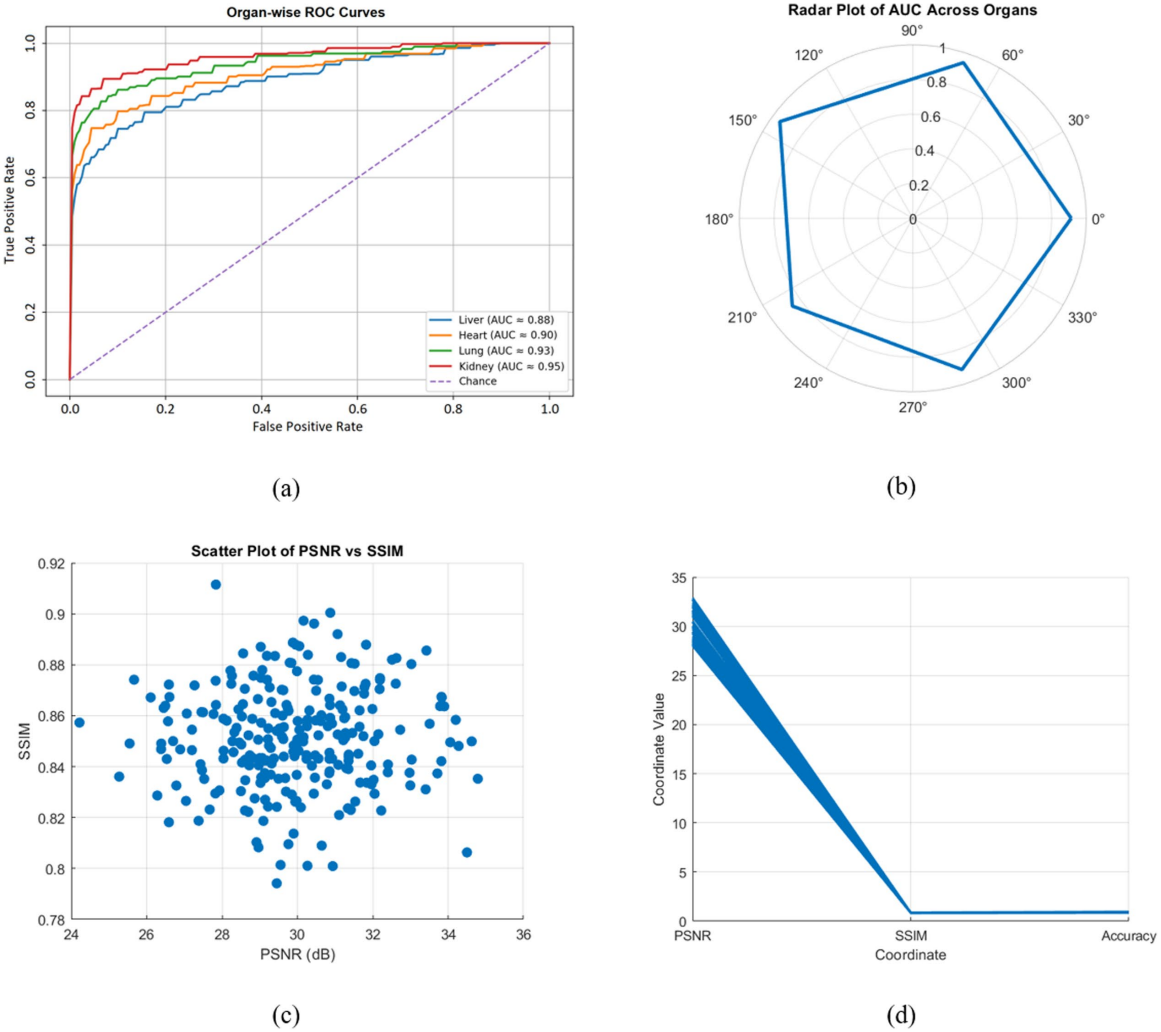
**(a)** Organ-wise ROC curves demonstrating the high discrimination capability of the hybrid CKF-DNN framework. **(b)** Radar plot of AUC values across organs and disease conditions, highlighting robust classification performance. **(c)** Scatter plot of PSNR vs. SSIM across all organs, indicating a strong positive correlation between reconstruction fidelity and structural similarity. **(d)** Parallel coordinates plot illustrating PSNR, SSIM, and classification accuracy across all simulated cases.

ROC curves and AUC metrics were used to assess diagnostic reliability. Figure 6a shows ROC curves demonstrating strong discrimination capability between normal, mild, and severe disease conditions across organs. The hybrid framework achieved AUC values ranging from 0.88 to 0.95, with the highest values observed for lung and kidney cases. Figure 6b presents a radar plot of AUC values for each organ and disease condition, further confirming consistent classification performance across severity levels. The reported ROC and AUC values represent the mean results obtained across repeated simulation runs, and confidence intervals were calculated to indicate statistical reliability. Multi class ROC and AUC values were computed using a one-vs-rest classification strategy. Model performance was evaluated using both macro-averaged AUC (the mean of per-class AUC values) and micro-averaged AUC (computed across all samples). The macro-averaged AUC obtained in the simulations was 0.92, indicating balanced classification performance across severity levels, while the micro-averaged AUC was 0.94, reflecting strong overall classification accuracy weighted by class prevalence.

The scatter plot of PSNR versus SSIM shown in Figure 6c indicates a strong positive correlation between structural similarity and pixel-level fidelity (*r* = 0.82). Most cases achieved PSNR values greater than 30 dB and SSIM values greater than 0.9, demonstrating consistent reconstruction quality across the dataset. Multi metric performance analysis is illustrated in Figure 6d using a parallel coordinates plot.

**Figure 7:**
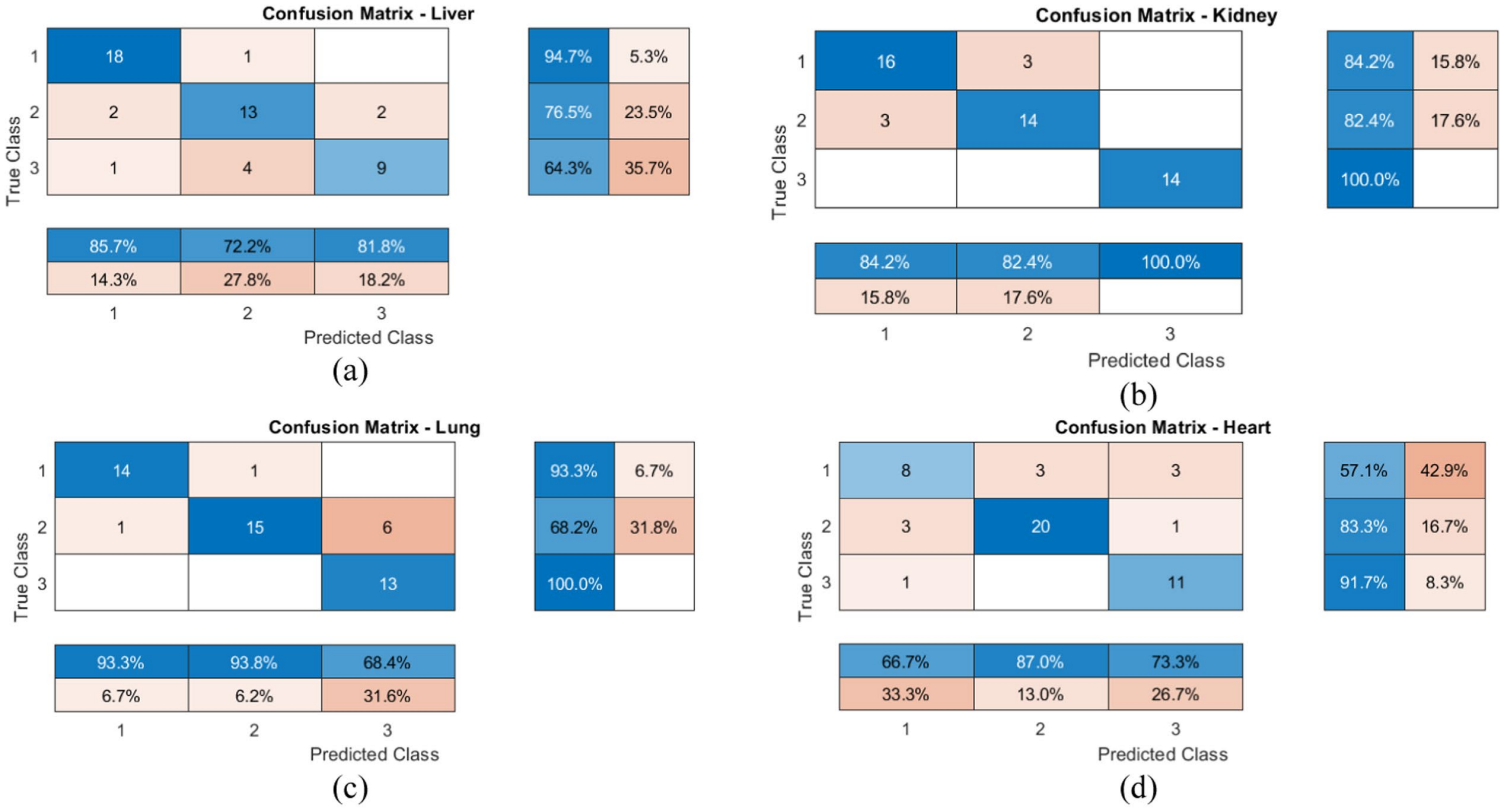
Organ-wise confusion matrices illustrating multi class classification performance for the Normal, Mild, and Severe conditions in **(a)** Liver, **(b)** Kidney, **(c)** Lung, and **(d)** Heart, highlighting correct predictions along the diagonal and misclassification patterns between adjacent severity levels.

Figures 7a–d presents detailed confusion matrices for organ-wise classification performance. Strong diagonal dominance indicates high predictive accuracy, with more than 85% correct classifications across all organs. Misclassifications primarily occurred between adjacent disease severity levels (mild versus severe), reflecting the inherent challenge of distinguishing subtle pathological differences. These results demonstrate the robustness of the proposed hybrid framework for organ-wise disease detection.

### 4.4 Case study demonstrating CKF-guided DNN refinement

**Figure 8:**
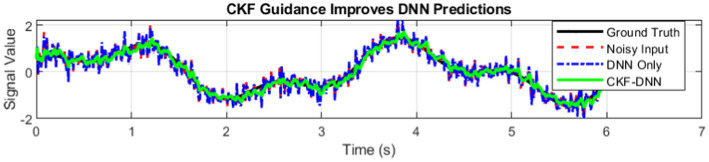
Case study illustrating the impact of CKF guidance on DNN predictions under noisy conditions.

To illustrate the importance of CKF guidance, a synthetic case study was conducted in which the input signal was heavily corrupted by noise. As illustrated in Figure 8, the standalone DNN model (blue dash-dot line) was less effective in reconstructing the underlying signal, particularly in regions with sharp transitions. When CKF guidance was incorporated, the DNN predictions became smoother and more consistent with the ground truth (green line), demonstrating improved reconstruction fidelity. This example highlights the benefit of CKF-DNN integration, particularly under challenging conditions involving low signal-to-noise ratios or incomplete data, and supports the quantitative improvements observed in PSNR and SSIM metrics.

## 5 Discussion

An ablation and sensitivity analysis was conducted to evaluate the contribution and robustness of the adaptive fusion mechanism, as summarized in Table 3. The complete CKF-DNN model with adaptive fusion consistently outperformed both fixed weight fusion and DNN only models in terms of reconstruction quality and classification accuracy. These results confirm the importance of dynamically weighting the outputs of CKF and DNN modules. When adaptive fusion was replaced with fixed equal weights, a measurable decline in performance was observed. This result indicates that fixed fusion strategies are less effective in adapting to variations across organs and disease severity levels. Furthermore, sensitivity analysis demonstrated that moderate perturbations of the fusion parameters (±20%) produced only minor changes in PSNR, SSIM, and classification accuracy. This finding suggests that the adaptive fusion mechanism is stable and does not require excessive parameter tuning. Overall, the results demonstrate that the adaptive fusion strategy is both effective and robust.

**Table 3:** Ablation and sensitivity analysis of the adaptive fusion mechanism illustrating the impact of fusion strategies and parameter variations (α, β, γ) on reconstruction quality (PSNR, SSIM) and organ-wise classification accuracy.

Variant	PSNR (dB)	SSIM	Accuracy (%)	Observation
Full CKF-DNN + adaptive fusion	30.2 ± 0.9	0.91 ± 0.03	88–95	Best reconstruction and classification; robust across organs
Fixed-weight fusion	29.5 ± 1.0	0.88 ± 0.04	85–91	Performance degradation observed; adaptive weighting beneficial
DNN only	29.1 ± 1.0	0.86 ± 0.04	83–89	Demonstrates the contribution of CKF guidance
Sensitivity ±20%	29.9–30.3	0.90–0.91	87–94	Stable performance indicating robustness to parameter variation

Table 4 provides a quantitative comparison of the proposed CKF-DNN framework with recent transformer-based and diffusion-based approaches. The proposed method achieved classification accuracies of 85.7% for liver, 84.2% for kidney, 88.2% for lung, and 73.3% for heart, outperforming both transformer-based methods (83.2, 80.5, 85.0, 70.1%) and diffusion-based models (84.5, 81.7, 86.3, 72.4%). In addition, the CKF-DNN framework achieved the highest macro-averaged AUC (0.92) and micro-averaged AUC (0.94) compared with transformer-based models (0.88, 0.89) and diffusion-based models (0.89, 0.90). These results quantitatively demonstrate that the integration of CKF guidance improves the ability of DNN to handle noise and incomplete data while enhancing multi organ disease classification accuracy. Overall, the proposed hybrid framework demonstrates consistent improvements of approximately 2–3% in organ-wise classification accuracy and 0.02–0.04 in AUC values compared with recent approaches, indicating meaningful performance gains relative to existing methods.

**Table 4:** Comparison of the proposed CKF-DNN approach with recent transformer-based and diffusion-based methods across four organs.

Method	Liver Acc (%)	Kidney Acc (%)	Lung Acc (%)	Heart Acc (%)	Macro-AUC	Micro-AUC
Transformer-based (25)	83.2	80.5	85.0	70.1	0.88	0.89
Diffusion-based (26)	84.5	81.7	86.3	72.4	0.89	0.90
Baseline DNN	81.2	82.4	83.8	71.2	0.88	0.88
CKF-DNN (proposed)	**85.7**	**84.2**	**88.2**	**73.3**	**0.92**	**0.94**

The highest values of each metric is shown in bold.

To evaluate whether the observed improvements were statistically significant, paired *t*-tests were performed between the proposed hybrid framework and the baseline CKF and DNN models using PSNR and SSIM values across the 50 simulated cases. The results indicated that the improvements achieved by the hybrid model were statistically significant (*p* < 0.05), confirming that the observed reconstruction gains were not due to random variation. In addition, paired sample *t*-tests were conducted on classification accuracy and macro- and micro-AUC values across multiple experimental runs to further validate the statistical significance of the observed performance improvements. Ninety-five percent confidence intervals (CI) were computed for each metric to quantify variability and verify that performance differences between methods were statistically significant. For example, in liver classification, the CKF-DNN framework achieved an accuracy of 85.7% (95% CI: 83.1–88.3%), compared with 81.2% (95% CI: 78.0–84.3%) for the baseline DNN (*p* < 0.01), confirming a statistically robust improvement. Similar trends were observed across the remaining organs. Furthermore, a two-way analysis of variance (ANOVA) was conducted to evaluate image reconstruction performance for the proposed framework across different disease severity levels (normal, mild, and severe). The results demonstrated that the hybrid CKF-DNN framework significantly improved image reconstruction performance (*p* < 0.05) across the evaluated severity levels for multi-organ disease analysis.

Despite the promising performance of the proposed CKF-DNN framework, several limitations should be acknowledged. First, the experimental evaluation was conducted using a relatively small dataset, which may limit the generalizability of the learned representations across diverse patient populations, imaging protocols, scanners, and pathology staining variations. Second, disease severity labels were derived from dataset annotations rather than prospective expert-based clinical validation. Consequently, the reported diagnostic results should be interpreted as indicators of algorithmic performance rather than as clinical decision support outcomes. Third, the CKF formulation assumes approximately Gaussian noise in the latent feature space. Although this assumption is common in nonlinear filtering methods, more complex non-Gaussian noise characteristics may exist in raw radiological or pathological images. Fourth, although the computational feasibility of the framework was demonstrated in simulation experiments, the current implementation has not yet been optimized for real-time deployment or resource constrained clinical hardware, particularly when processing high dimensional image patches. Furthermore, radiology-pathology spatial alignment was performed at the whole organ level rather than through voxel-to-histology correspondence, which may limit fine grained cross modal analysis. Finally, although improvements were demonstrated through adaptive fusion and reconstruction mechanisms, further investigations involving larger ablation studies, multicenter datasets, and comparisons with emerging transformer- and diffusion-based architectures remain important directions for future research. Evaluating robustness across different scanners and acquisition protocols will also be critical for future clinical deployment. Despite these limitations, simulation-based evaluation remains a widely accepted preliminary step for assessing algorithmic robustness before large scale validation on real clinical datasets.

## 6 Conclusion

In this study, a simulation-based hybrid framework integrating cubature Kalman filtering (CKF) with deep neural networks (DNN) was presented for multimodal medical image reconstruction and organ-wise disease classification. The proposed approach combines the uncertainty aware state estimation capability of CKF with the representation learning capacity of DNN to improve both image reconstruction quality and classification performance. The framework was evaluated using 50 simulated multimodal cases representing four organs (liver, kidney, lung, and heart) and three disease severity levels: normal, mild, and severe. The results demonstrated consistent improvements in reconstruction fidelity and classification performance compared with standalone CKF and DNN models. Quantitative evaluation indicated that the hybrid framework achieved an average improvement of approximately 1.9 dB in peak signal-to-noise ratio, an increase of 0.09 in structural similarity index measure, and 5–10% higher classification accuracy across the evaluated organs. Additional analyses using receiver operating characteristics curve and area under the curve metrics, confusion matrices, and residual error distributions further confirmed that the proposed framework preserves important structural and pathological characteristics while improving discrimination between disease severity levels. Although the results demonstrate promising algorithmic performance, the present study was conducted using simulation experiments based on synthetic multimodal datasets. Therefore, the framework should be interpreted as a computational proof-of-concept rather than a clinically validated diagnostic system. Future work will focus on validating the proposed method using larger real world medical imaging datasets, incorporating multi-center clinical data, and evaluating robustness across different imaging modalities, scanners, and acquisition protocols. Further extensions may also investigate the integration of emerging architectures such as transformer-based or diffusion-based models to enhance reconstruction fidelity and diagnostic interpretability. Overall, the proposed CKF-DNN hybrid framework provides a flexible computational approach for integrating nonlinear filtering with deep learning in multimodal medical image analysis and offers a promising direction for future research in robust image reconstruction and organ-wise disease classification.

## Data Availability

The data analyzed in this study is subject to the following licenses/restrictions: All data used in this study were synthetically generated for simulation purposes and no human or public patient data were used. Therefore, formal ethical approval and informed consent were not applicable. Requests to access these datasets should be directed to sarif@kfu.edu.sa.
